# Ultrasound Blood-Brain Barrier Opening and Aducanumab in Alzheimer’s Disease: A Systematic Review and Meta-Analysis

**DOI:** 10.7759/cureus.68008

**Published:** 2024-08-28

**Authors:** Noha S Aljehani, Seba T Al-Gunaid, Alhassan H Hobani, Mohammad F Alhinti, Yahya A Khubrani, Lama M Abu-Hamoud, Aljawharah A Alrayes, Lama B Alharbi, Ali A Sultan, Doaa A Turkistani, Seddiqa S Naiser, Leen Albraik, Amjad M Alakel, Muteb Alotaibi, Abdullah Y Asiri

**Affiliations:** 1 College of Medicine, Jazan University, Jazan, SAU; 2 College of Medicine, Syiah Kuala University, Banda Aceh, IDN; 3 College of Medicine, Imam Mohammad Ibn Saud Islamic University, Riyadh, SAU; 4 Applied Medical Science and Diagnostic Radiography Technology, Albaha Health Cluster, Albaha, SAU; 5 College of Pharmacy, Taibah University, Madinah, SAU; 6 College of Medicine, Jouf University, Jouf, SAU; 7 College of Medicine, University of Jeddah, Jeddah , SAU; 8 College of Medicine, King Khalid University, Abha, SAU; 9 College of Medicine, Batterjee Medical College, Jeddah, SAU; 10 College of Medicine, Xi’an Jiaotong University, Xi’an, CHN; 11 College of Medicine, Alfaisal University, Riyadh, SAU; 12 College of Medicine, King Saud Bin Abdulaziz University for Health Sciences, Riyadh, SAU

**Keywords:** blood-brain barrier opening, blood-brain barrier, ultrasound, aducanumab, alzheimer’s disease

## Abstract

The blood-brain barrier (BBB) presents a significant challenge in treating Alzheimer's disease, as it restricts the delivery of therapeutic medications to brain tissue. Reversible breaking of the BBB using low-intensity focused ultrasound guided by magnetic resonance imaging (MRI) may benefit patients with Alzheimer's disease and other neurological illnesses, such as brain tumors, amyotrophic lateral sclerosis, and Parkinson's disease. This systematic study and meta-analysis aimed to assess aducanumab and the ultrasonography of BBB opening in Alzheimer's patients. According to Preferred Reporting Items for Systematic Reviews and Meta-Analyses (PRISMA), the study was conducted by searching six digital repositories for relevant scholarly literature, focusing on English papers published between 2015 and 2024; the data was extracted using an Excel sheet, and data was analyzed using Revman 5.4.1 software. The study's findings indicate that the groups receiving ultrasound and aducanumab treatment benefited from it; however, overall, the effect was not statistically significant (P=0.29) at 95% CI 0.86 (0.75, 1.00). With regard to side effects, the results indicate that the treatment had fewer side effects compared to the control group; however, the difference was not statistically significant (p=0.94) at 95% CI 0.93 (0.70, 1.22). The study found a positive effect of ultrasound and aducanumab on the treatment groups, but it was not statistically significant. The control group had less side effects than the treatment group. Therefore, future studies should focus on the quantity or combination of the drug that yields more effective results.

## Introduction and background

Alzheimer's disease (AD) is a progressively worsening brain disorder that significantly impacts memory, cognitive functions, and overall quality of life [1]. AD was first described by Alois Alzheimer as a horrible cortical disease when he found significant neuronal loss and amyloid plaques in the brain of a patient who suffered from personality changes and memory loss [2]. One of the major hurdles in treating AD has been the restricted ability of therapeutic drugs to penetrate the blood-brain barrier (BBB) [3].

 The BBB can be temporarily disrupted in patients with AD and other neurological conditions, such as brain tumors, amyotrophic lateral sclerosis, and Parkinson's disease through the application of low-intensity focused ultrasound guided by magnetic resonance imaging (MRI) [4,5]. However, recent advancements in medical technology have shown promise in addressing this challenge. Low-intensity focused ultrasound, guided by MRI, offers a novel approach to temporarily and reversibly open the BBB [3]. This breakthrough allows for more efficient delivery of therapeutic agents, such as aducanumab, directly to affected brain regions in AD patients [4]. Aducanumab, an anti-amyloid antibody, targets the amyloid-beta (Aβ) plaques that are characteristic of AD pathology, aiming to reduce their accumulation and potentially slow disease progression [5].

Previous studies using targeted ultrasound to particular brain regions in individuals with AD without administering a therapeutic intervention have shown results with somewhat decreased levels of Aβ in some places [6,7]. There is evidence that anti-Aβ antibodies can reduce Aβ levels and postpone the start of disease [8]. By using focused ultrasound to penetrate the BBB, aducanumab distribution to specified brain regions was five to eight times higher in animal models than in brain regions that were not treated [9,10]. Aiming to evaluate the feasibility of aducanumab infusion and the impact of opening the BBB via ultrasonography on the removal of amyloid in AD, the prospective, single-group, single-institution, open-label proof-of-concept trial was started by the investigator, involving three participants [11,12].

In order to provide recommendations for the use of both ultrasound BBB opening and aducanumab among AD patients, this paper reviews the data that is currently available on these two therapies. It also aims to compile the main points of contention surrounding the regulatory bodies' evaluation of aducanumab. A systematic review and meta-analysis of the various studies are used to come up with the conclusion and the recommendations.

## Review

Material and methods

The research adhered to the guidelines set forth by Preferred Reporting Items for Systematic Reviews and Meta-Analyses (PRISMA) and searched six digital repositories for relevant scholarly articles published between 2015 and 2024, which include MEDLINE, PUBMED, Google Scholar, Cochrane Library, EMBASE, and Web of Science [13]. The combinations of keywords utilized in the search for articles were “Alzheimer’s disease, aducanumab, ultrasound blood-brain barrier, and blood-brain barrier opening.” The study protocol was registered on the Prospective Register of Systematic Reviews (PROSPERO) database (registration no: CRD42024565313).

Eligibility, Data Extraction, and Management

Criteria for inclusion: Peer review and English publication dated from January 2015 are prerequisites for inclusion. Included studies that make use of rigorous research methods (such as case-control studies, randomized, double-blind, placebo-controlled, and comparative studies) and that include individuals who have received AD treatment. This review includes studies done anywhere in the world, regardless of how severe AD is or how it affects individuals, with mild AD dementia or moderate cognitive impairment (MCI).

Studies excluded: Studies that are not directly related to the study question or that do not report results for the therapy of AD. This category includes research that has not been published in English, duplicate studies, studies with incomplete data or reporting, studies with serious methodological flaws or a high risk of bias, and studies that are irrelevant or only slightly further the objectives of the review. The evaluation aims to guarantee the inclusion of pertinent, high-caliber research that offers significant new insights regarding aducanumab and the opening of the ultrasonography BBB in individuals with AD by utilizing these exclusion criteria.

Based on the predetermined criteria, the studies were independently examined and chosen by two seasoned researchers. The researcher followed the PRISMA criteria to the letter, and disagreements were discussed and resolved [14]. Ultimately, the researcher extracted data from the selected studies and recorded it.

Statistical Data Analysis

The analysis used Revman 5.4.1 software to compute metrics like risk ratio, random effect, standard error (SE), and CIs for event-related studies. Multivariate random-effects meta-regression was used to quantify heterogeneity, and I² values were classified into four quartiles: low, low-to-moderate, moderate-to-high, and high heterogeneity, ranging from 0% to <25%, 25% to <50%, and >75%, respectively. The quality of items risk of bias assessment summary bar graph and funnel plot asymmetry test were used to quantify publishing and item bias. It was determined that each analysis was statistically significant at the 0.05 level.

Results

**Figure 1:**
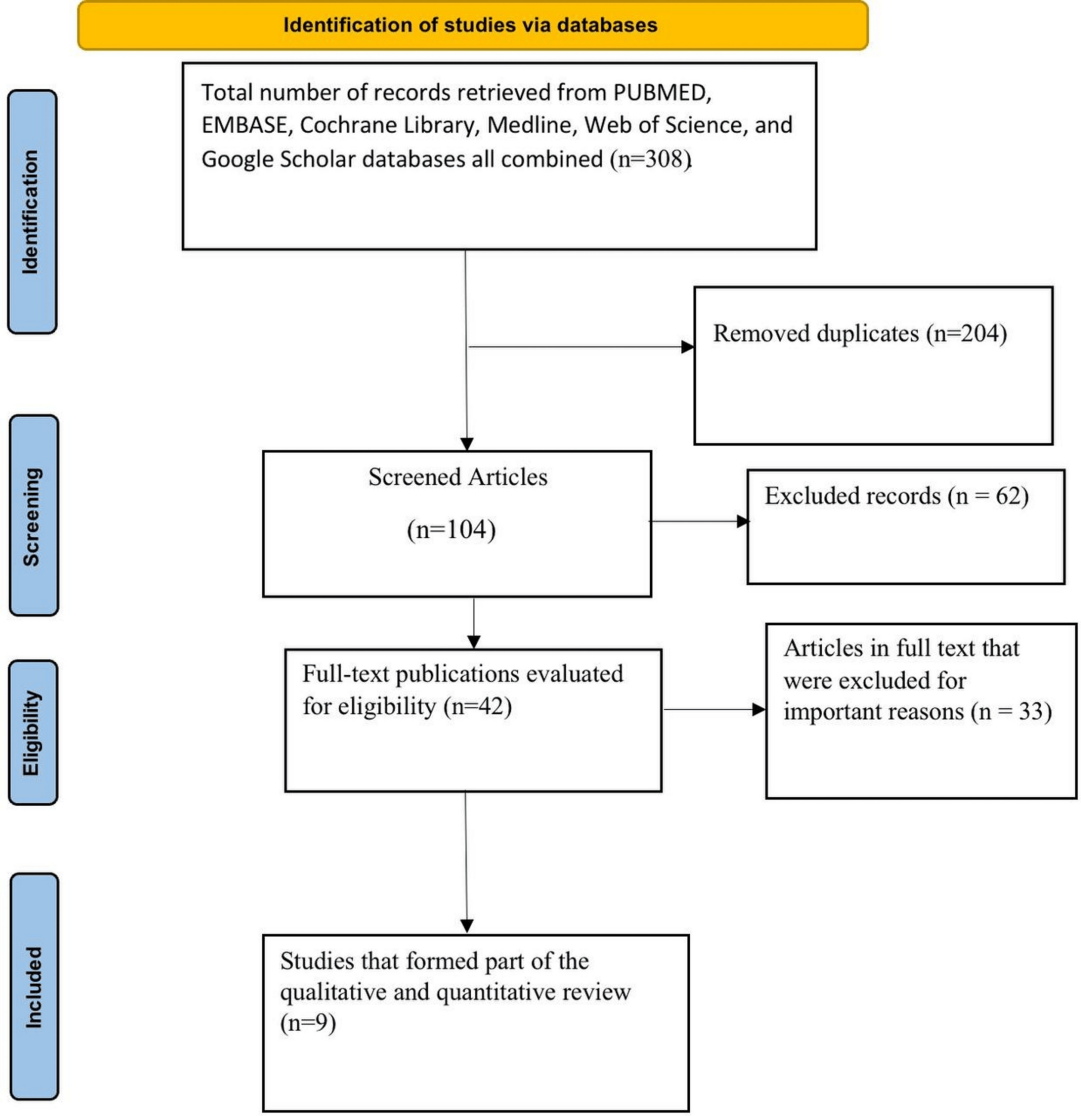
PRISMA flow diagram PRISMA, Preferred Reporting Items for Systematic Reviews and Meta-Analyses

Using the search strategy previously outlined in the methodology, 308 articles were found in the various databases: PubMed showed 54, Embase showed 51, the Cochrane Library showed 50, Medline showed 52, Web of Science showed 53, and Google Scholar showed 48 article results. The predetermined inclusion and exclusion criteria were followed in order to assess and analyze the eligibility of 14 records before they were finally included in the systematic review. Figure 1 of the PRISMA flow diagram illustrates this.

Study Characteristics

The key features of the studied research are shown in Table 1. The nine articles that made up this systematic review discuss aducanumab and the opening of the ultrasound BBB in Alzheimer's patients. The nine investigations were published in English and were conducted in different parts of the world. Furthermore, an AD intervention was discussed in all nine articles. 6703 subjects made up the entire sample size across the nine investigations. The distinctive features of each study are presented across the rows, while the general research attributes of each column are given beneath it.

**Table 1:** Characteristics of the included studies AD, Alzheimer's disease; BBB, blood-brain barrier; SAEs, severe adverse events; Aβ, amyloid-beta; ARIA, amyloid-related imaging abnormalities (ARIA)

Authors	Region	Sample size	Study design	Intervention	Inclusion	Outcome/results
Haeberlein et al., 2022 [15]	Multiple countries: Canada, UK, USA, and Sweden	1812	Randomized, double-blind, placebo-controlled	Aducanumab	Clinical trials, RCTs, case-controlled trials, and studies involving ultrasound or Aducanumab in treatment of AD	Target engagement and a dose-dependent decrease in pathophysiological indicators of AD were validated by the results of biomarker substudies. Imaging abnormalities associated with amyloid-induced edema were the most frequent adverse event.
Ferrero et al., 2016 [16]	USA and Switzerland	53	Randomized, double-blind, placebo-controlled	Aducanumab	Clinical trials, RCTs, case-controlled trials, and studies involving ultrasound or Aducanumab in treatment of AD	Tolerability and safety were the main results. In general, doses ≤30 mg/kg were well tolerated, and there were no moderate or SAEs. After receiving 60 mg/kg aducanumab, all three patients experienced symptomatic ARIA (SAEs), which went away entirely by weeks 8-15.
Vaz et al., 2022 [17]	Portugal	706	Clinical trials	Aducanumab	Clinical trials, RCTs, case-controlled trials, and studies involving ultrasound or aducanumab in treatment of AD	The ability of aducanumab to impact downstream tau pathology may pave the way for the use of combination therapies (drugs that target both tau and amyloid) to treat AD.
Plowey et al., 2022 [18]	USA	1	Clinical case-controlled trial	Aducanumab	Clinical trials, RCTs, case-controlled trials, and studies involving ultrasound or Aducanumab in treatment of AD	According to the study, aducanumab lessens the neuropathology caused by Aβ plaque in AD patients.
Logovinsky et al., 2016 [19]	Sweden	40	A multicenter, randomized, double-blind, placebo-controlled investigation	Ultrasound	Clinical trials, RCTs, case-controlled trials, and studies involving ultrasound or Aducanumab in treatment of AD	ARIA-E/H had an MRI incidence that was comparable to a placebo. With a half-life of roughly seven days for serum terminal elimination, the exposure to BAN2401 was roughly dose-proportional. There was only a minor rise in plasma Aβ, while BAN2401 had no discernible effects on CSF biomarkers.
Haeberlein et al., 2020 [20]	USA	3285	Randomized, double-blind, placebo-controlled	Aducanumab	Clinical trials, RCTs, case-controlled trials, and studies involving ultrasound or Aducanumab in treatment of AD	A substantial reduction in CDR-SB scores was observed in patients 78 weeks following high doses of aducanumab (22% versus placebo, P=0.01).
Kuller et al., 2021 [21]	USA	603	Clinical trials	Aducanumab	Clinical trials, RCTs, case-controlled trials, and studies involving ultrasound or Aducanumab in treatment of AD	Both the high-dose EMERGE experiment and the ENGAGE trial originally yielded no benefits, but after a longer follow-up period, a notable beneficial advantage was observed.
O'Gorman et al., 2017 [22]	Spain	196	Clinical trials	Aducanumab	Clinical trials, RCTs, case-controlled trials, and studies involving ultrasound or Aducanumab in treatment of AD	Acelananumab has demonstrated satisfactory safety and tolerability in lowering Aβ plaques and delaying the clinical measure decrease in prodromal or mild AD patients. ARIA were the main safety observation. A dose-dependent adverse effect associated with the removal of Aβ, ARIA was more prevalent in ApoE ε4 carriers than in non-carriers.
Lipsman et al., 2018 [23]	Canada	5	Clinical controlled trials	Ultrasound	Clinical trials, RCTs, case-controlled trials, and studies involving ultrasound or Aducanumab in treatment of AD	The BBB was opened repeatedly, safely, and reversibly within the intended volume. There was no major clinical or radiological adverse event linked to breaching the BBB at three months, nor was there a clinically significant reduction in cognitive scores from baseline values.

Assessment of Item Risk of Bias of the Studies

**Figure 2:**
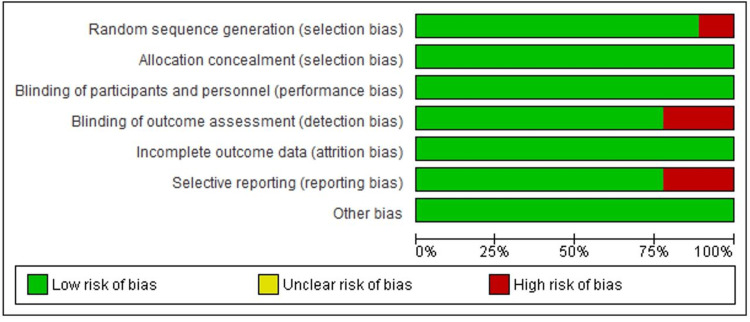
Summary of studies’ risk of bias of each item

Figure 2 demonstrates that most of the included papers had low bias risk and were of excellent quality. To remove prejudice in performance, reporting, attrition, detection, and selection, a random sequence generator was used. However, the data indicates that there is a high risk of detection bias in 2/9, a high risk of reporting bias in 2/9, and a high risk of selection bias in 1/9 [15-18,22].

Funnel Plot of the Publication Bias

**Figure 3:**
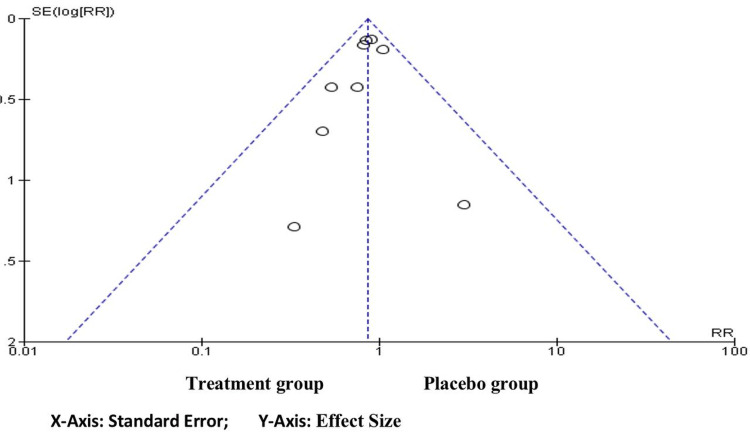
Funnel plot depicting publication bias RR, relative risk; SE, standard error

A symmetric distribution of impact sizes shows how research precision changes in Figure 3. The funnel plot with an unequal number of studies on each side is depicted, with more studies aligned to the left. This suggests that there's a chance that the treatment group was favored in publications.

Meta-Analysis of the Studies Outcome

**Figure 4:**
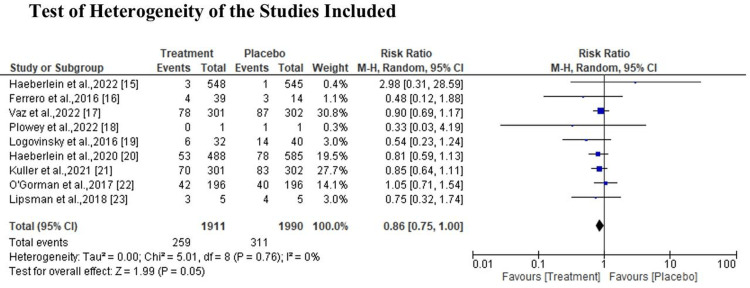
Effect and side effect of ultrasound BBB opening and aducanumab Sources: Haeberlein et al., 2022; Ferrero et al., 2016; Vaz et al., 2022; Plowey et al., 2022; Logovinsky et al., 2016; Haeberlein et al., 2020; Kuller et al., 2021; O'Gorman et al., 2017; Lipsman et al., 2018 [15-23]. BBB, blood-brain barrier

**Figure 5:**
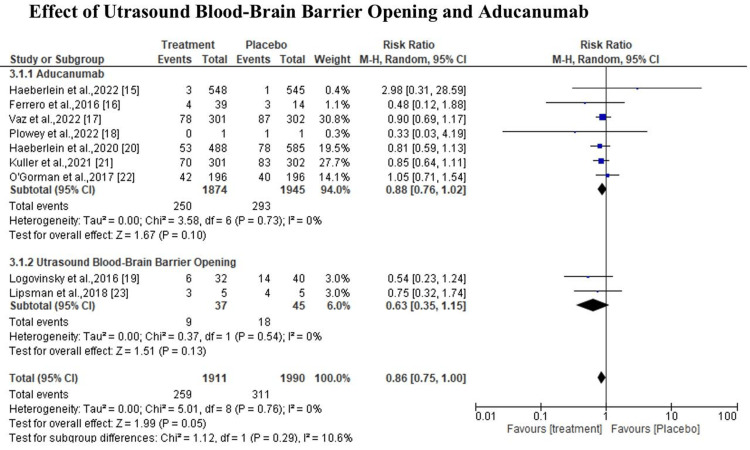
Effect and side effect of ultrasound BBB opening and aducanumab Sources: Haeberlein et al., 2022; Ferrero et al., 2016; Vaz et al., 2022; Plowey et al., 2022; Logovinsky et al., 2016; Haeberlein et al., 2020; Kuller et al., 2021; O'Gorman et al., 2017; Lipsman et al., 2018 [15-23]. BBB, blood-brain barrier

**Figure 6:**
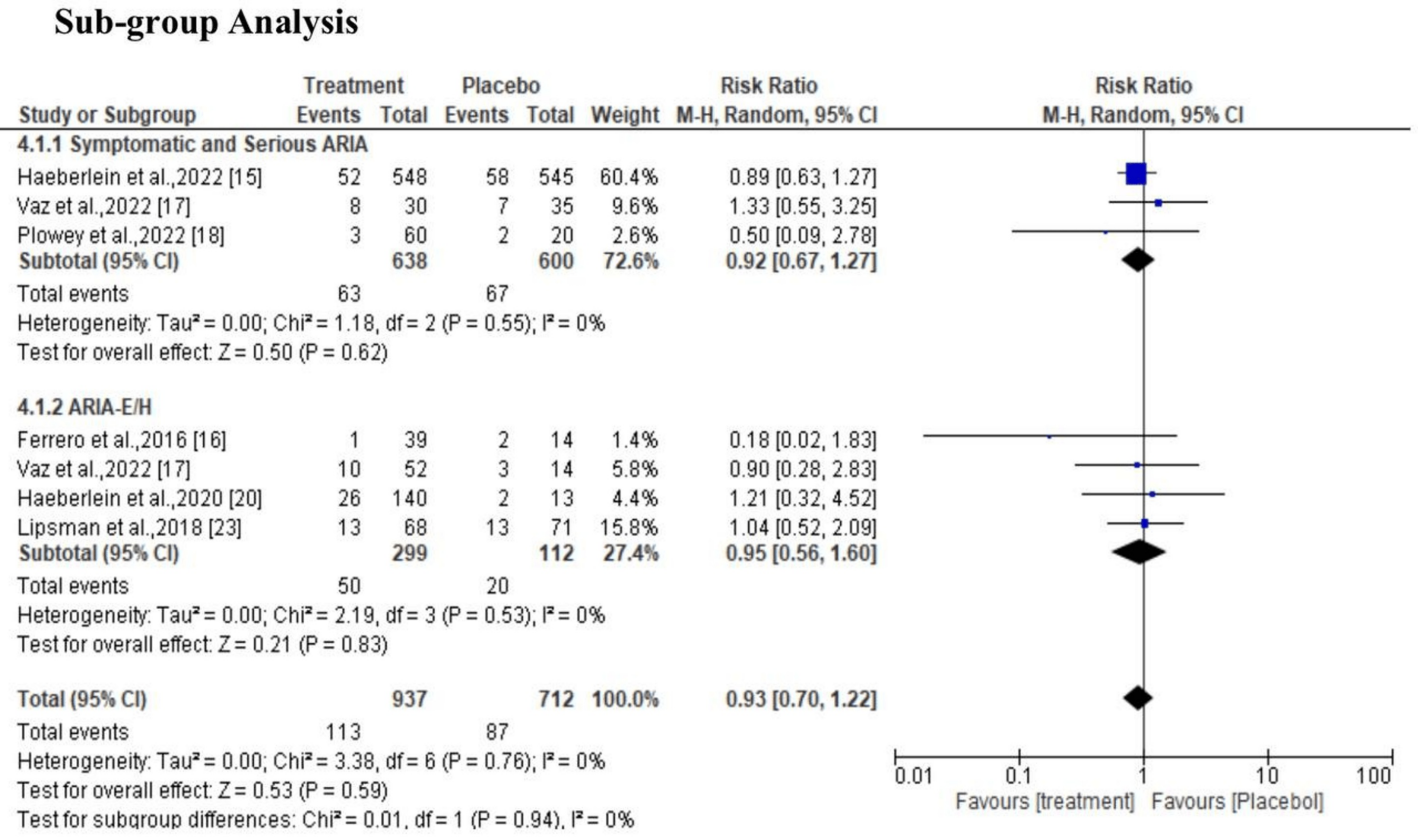
Effect and side effect of ultrasound BBB opening and aducanumab Sources: Haeberlein et al., 2022; Ferrero et al., 2016; Vaz et al., 2022; Plowey et al., 2022; Logovinsky et al., 2016; Haeberlein et al., 2020; Kuller et al., 2021; O'Gorman et al., 2017; Lipsman et al., 2018 [15-23]. BBB, blood-brain barrier

The heterogeneity of the studies used was not statistically significant, I2=0%, X2=5.01 at 95% CI 0.86 (0.75, 1.00) (P=0.76) (Figure 4). According to sub-group analysis, the diamond marker shows there was a positive effect in favor of the groups treated with ultrasound and aducanumab; however, the overall effect was not statistically significant (P=0.29) at 95% CI 0.86 (0.75, 1.00) (Figure 5). In regards to side effects, the results indicate that the treatment had fewer side effects compared to the control group; however, the difference was not statistically significant (p=0.94) at 95% CI 0.93 (0.70, 1.22) (Figure 6).

Discussion

This systematic review and meta-analysis focused on evaluating the ultrasound BBB opening and aducanumab in AD. The study finding reveals that the heterogeneity of the studies used was not statistically significant, I2=0%, X2=5.01 at 95% CI 0.86 (0.75, 1.00) (P=0.76). As noted in a study by Haeberlein et al., comparing the drug-treated groups to the placebo groups, the former displayed noticeably more common amyloid-related imaging abnormalities (ARIA), such as edema, hemorrhage, and symptomatic and severe ARIA. Even after 18 months, there is disagreement over the level of risk associated with high-clearance immunotherapies in the early stages of AD. Their therapeutic value may be increased by determining subgroups of better responders, examining combination therapy from a different angle, and doing a longer follow-up [15]. Furthermore, a study by Ferrero et al., noted that there were no moderate or severe adverse events (SAEs). After receiving 60 mg/kg aducanumab, all the patients experienced symptomatic ARIA (SAEs), which went away entirely by weeks 8-15 [16].

In addition, the finding of the study indicates that there was a positive effect in favor of the groups treated with ultrasound and aducanumab; however, the overall effect was not statistically significant (P=0.29) at 95% CI 0.86 (0.75, 1.00). As noted in a study by Vaz et al., the ability of aducanumab to impact downstream tau pathology may pave the way for the use of combination therapies (drugs that target both tau and amyloid) to treat AD [17]. Furthermore, according to the study by Ploweyet al., aducanumab lessens the neuropathology caused by Aβ plaque in AD patients [18]. However, Logovinsky et al., noted in their study that on MRI, the incidence of ARIA-E/H was similar to that of a placebo. There was only a minor rise in plasma Aβ, while BAN2401 had no significant effects on CSF biomarkers [19].

With regard to side effect, the results indicate that the control had fewer side effect compared to the treatment group; however, the difference was not statistically significant (p=0.94) at 95% CI 0.93 (0.70, 1.22). This was consistent with the finding in a study by Haeberlein et al., which revealed that after receiving high doses of aducanumab, patients' CDR-SB scores at 78 weeks slightly decreased from baseline (22% versus placebo, P = 0.01) [20]. More so, Kuller et al. noted in their study that both the high-dose EMERGE experiment and the ENGAGE trial originally yielded no benefits in a short period of follow-up, but after a longer follow-up period, a notable beneficial advantage was observed [21]. However, O'Gorman et al. revealed in their study that acelananumab demonstrated satisfactory safety and tolerability in lowering Aβ plaques and delaying the clinical measure decrease in prodromal or mild AD patients. The primary safety observation was ARIA, a dose-dependent side effect linked to the elimination of Aβ that was more common in ApoE ε4 carriers than in non-carriers [22]. On the other hand, a study by Lipsman et al. noted that there was no major clinical or radiological adverse event linked to breaching the BBB at three months nor was there a clinically significant reduction in cognitive scores from baseline values [23].

This study was limited in some ways. The study did not focus on the quantity of aducanumab viable for the treatment of AD, thus it is difficult to tell the effectiveness of the drug in the treatment of the disease. Moreover, AD is more common as people age; therefore, a lack of focus on a specific age group would have further hampered the generalizability of the study findings.

## Conclusions

The study found a positive effect of ultrasound and aducanumab on the treatment groups, but it was not statistically significant. Furthermore, the study found that the control group had fewer side effects on the patients than the treatment group. Therefore, future studies should focus on the quantity or combination of the drug that yields more effective results and that is safe from the side effects revealed in this study.
